# Spot Scanning Proton Therapy for Sinonasal Malignant Tumors

**DOI:** 10.14338/IJPT-D-20-00043.1

**Published:** 2021-06-25

**Authors:** Koichiro Nakajima, Hiromitsu Iwata, Yukiko Hattori, Kento Nomura, Shingo Hashimoto, Toshiyuki Toshito, Kensuke Hayashi, Yo Kuroda, Hideo Fukano, Hiroyuki Ogino, Yuta Shibamoto

**Affiliations:** 1 Department of Radiation Oncology, Nagoya Proton Therapy Center, Nagoya City West Medical Center, Nagoya, Japan; 2 Department of Radiology, Nagoya City University Graduate School of Medical Sciences, Nagoya, Japan; 3 Department of Proton Therapy Physics, Nagoya Proton Therapy Center, Nagoya, Japan; 4 Department of Proton Therapy Technology, Nagoya Proton Therapy Center, Nagoya City West Medical Center, Nagoya, Japan; 5 Department of Otorhinolaryngology, Nagoya City West Medical Center, Nagoya, Japan; 6 Department of Oral and Maxillofacial Surgery, Nagoya City West Medical Center, Nagoya, Japan

**Keywords:** proton therapy, spot scanning, sinonasal cancer

## Abstract

**Purpose:**

Treatment of sinonasal malignant tumors is challenging, and evidence to establish a standard treatment is limited. Our objective was to evaluate the efficacy and safety of spot scanning proton therapy (SSPT) for sinonasal malignant tumors.

**Patients and Methods:**

We retrospectively analyzed patients with sinonasal malignant tumors (T1-4bN0-2M0) who underwent SSPT between May 2014 and September 2019. The prescription dose was typically either 60 GyRBE in 15 fractions or 60.8 GyRBE in 16 fractions for mucosal melanoma and 70.2 GyRBE in 26 fractions for other histologic subtypes. Endpoints included local control (LC), progression-free survival, overall survival (OS), and incidence of toxicity. Prognostic factors were analyzed using the Kaplan-Meier method and log-rank test.

**Results:**

Of 62 enrolled patients, the common histologic subtypes were mucosal melanoma (35%), squamous cell carcinoma (27%), adenoid cystic carcinoma (16%), and olfactory neuroblastoma (10%). Locally advanced stages were common (T3 in 42% and T4 in 53%). Treatment-naïve tumors and postsurgical recurrent tumors accounted for 73% and 27%, respectively. No patient had previous radiotherapy. The median follow-up was 17 months (range, 6-66) for all patients and 21.5 months (range, 6-66) for survivors. The 2-year LC, progression-free survival, and OS rates of all patients were 92%, 50%, and 76%, respectively. Univariate analysis revealed histology as a prognostic factor for OS, being higher in adenoid cystic carcinoma and olfactory neuroblastoma than in other tumors. Sixteen grade ≥3 late toxicities were observed in 12 patients (19%), including 11 events resulting in visual impairment; the most common was cataract. There was 1 grade 4 toxicity, and there were no grade 5 toxicities.

**Conclusion:**

SSPT was well tolerated and yielded good LC for sinonasal malignant tumors. Although we consider SSPT to be a leading treatment modality, further studies are required to establish its status as a standard treatment.

## Introduction

Sinonasal malignant tumors originating from the nasal cavity and paranasal sinuses are relatively rare, accounting for only about 3% of cancers in the upper respiratory and digestive tract [1]. The appropriate treatment strategy for sinonasal malignant tumors remains controversial, and existing treatment guidelines are supported only by weak clinical evidence [2]. This limited evidence has highlighted the importance of multimodal therapy consisting of surgery, radiation therapy (RT), and systemic therapy [3–5].

Although there have been no randomized clinical trials for sinonasal tumors, mainly because of their rarity and histologic heterogeneity [1, 6], several retrospective studies regarding RT have been conducted [7–9]. RT to sinonasal tumors is usually challenging because these tumors are often in close proximity to critical healthy tissues, such as the eyes, optic pathway, brain, and brainstem. In addition, some histologic subtypes are known to be radioresistant, for example, mucosal melanoma (MM), adenoid cystic carcinoma, adenocarcinoma, and olfactory neuroblastoma [10–12]. However, recent technological advances in RT have improved outcomes with regard to tumor control and safety [13–15]. Proton therapy (PT) is considered an especially promising treatment option. Indeed, both planning studies and clinical studies have suggested the superiority of PT over conventional photon therapy, including intensity-modulated radiotherapy [14, 15].

Spot scanning proton therapy (SSPT) is a pencil beam scanning (PBS) technique and is currently a predominant delivery system for PT, alongside passively scattered proton therapy (PSPT) [16]. In theory, SSPT, especially intensity-modulated proton therapy (IMPT), permits a more conformal dose distribution than PSPT [17, 18]. SSPT is considered to be advantageous for challenging cases requiring highly complex plans and may therefore be appropriate for the treatment of sinonasal tumors. SSPT is clearly becoming the mainstream of PT, with almost all recently constructed and planned PT facilities using SSPT [19]. However, the published evidence regarding SSPT for sinonasal tumors remains limited [20]. The present study aimed to investigate the clinical outcomes of SSPT for sinonasal malignant tumors.

## Patients and Methods

### Study Design and Patient Eligibility

We conducted a single-institutional retrospective analysis of patients with sinonasal malignant tumors who received SSPT between May 2014 and September 2019 at Nagoya Proton Therapy Center. This study was approved by our institutional review board (approval number: 20-04-311-01). The eligibility criteria were (1) histologically confirmed nasal cavity or paranasal sinus malignant tumors, (2) no evidence of distant metastasis, (3) no previous radiotherapy around the lesion, and (4) a follow-up duration ≥6 months for survivors. Before treatment, all eligible patients provided written informed consent, including agreement to possible use of their data for research purposes. The primary endpoint of the study was local control (LC). In addition, we also analyzed regional control (RC), distant metastasis-free survival (DMFS), progression-free survival (PFS), overall survival (OS), and incidence of acute and late toxicities.

### Simulation

Treatment simulation for PT was performed with noncontrast 1-mm-thick computed tomography (CT) images (Aquilion LB, Canon Medical Systems, Tochigi, Japan). Patients were immobilized in the supine position using a customized thermoplastic mask. A bite block was used to stabilize the mandible and deflect the tongue to the outside of the treatment volume. Additionally, before simulation, dental metal crowns and root canal fillings were replaced by low-density composites as much as possible to minimize dose calculation uncertainties.

### Target Delineation and Prescription

Target volumes and organ-at-risk structures were contoured by radiation oncologists. Magnetic resonance imaging was performed within a few days before or after CT simulation in all patients using a 1.5-T system (Magnetom Avanto, Siemens Healthineers, Erlangen, Germany). Axial noncontrast 5-mm-thick T1- and T2-weighted turbo spin echo images and diffusion-weighted images were obtained. In addition, axial contrast-enhanced 1-mm-thick T1-weighted fat saturated images were obtained to make fusion with CT images. ^18^F-deoxyglucose-positron emission tomography-CT (FDG-PET/CT) were used whenever possible to assist in target delineation. For postoperative cases, preoperative imaging data were also referenced. The gross tumor volume included the radiographically and/or endoscopically visible primary lesions plus, when present, positive lymph nodes. The clinical target volume (CTV) delineation was adjusted based on the tumor histology. In general, except for MM, the CTV margin to the gross tumor volume ranged between 3 and 5 mm, and was slightly adjusted depending on the surrounding anatomy. For MM, the CTV margin was expanded up to 10 mm only to the mucosal region. Prophylactic regional lymph node irradiation was not given. The planning target volume was designated by expansion of the CTV using the concept of beam-specific planning target volume reported by Park et al [21] with a 3-mm setup error and ±3.5% range uncertainties. The prescription dose was typically either 60 GyRBE in 15 fractions or 60.8 GyRBE in 16 fractions for MM, and 70.2 GyRBE in 26 fractions for other tumors, but 1 patient with olfactory neuroblastoma received 70 GyRBE in 35 fractions and another with malignant solitary fibrous tumor received 65 GyRBE in 26 fractions. The prescribed PT doses used a relative biological effectiveness value of 1.1 [22].

### Treatment Planning and Delivery

PT treatments were planned with VQA (Hitachi, Ltd, Tokyo, Japan) and delivered by PROBEAT III (Hitachi, Ltd), as previously described [23]. All patients were treated using the spot-scanning technique. For dose calculation of the scanning beams, the VQA system uses a pencil beam algorithm with a fluence-dose model to handle the aperture [24]. We initially used single-field optimization (SFO) for SSPT planning. Subsequently, multi-field optimization (MFO) became available at our institution from August 2016. In MFO planning, the worst-case optimization method has been applied to improve the robustness of treatment plans [25]. Thus, after both became available, comparable plans with both SFO and MFO were created for each case, and the better plan was selected. Hereafter, SSPT with SFO is referred to as single-field uniform dose (SFUD) and SSPT with MFO as IMPT. The beam energies were 70-180 MeV, and the spot size of 1 sigma ranged from 6 to 14 mm. Additionally, we implemented a patient-specific aperture system (PSAS) with attached energy absorber and collimator to reduce the lateral penumbra and improve the dose distribution. This system was previously reported in detail [26, 27].

During PT, daily pretreatment orthogonal 2-dimensional kV radiographic images were obtained to ensure precise patient positioning. Moreover, a verification CT scan was typically taken at least once during the treatment course to assess interfractional changes in anatomy and tumor volume that might affect the dose distribution, and replanning was performed when considered desirable.

### Evaluation of Clinical Outcome

During PT, patients were examined at least once a week by a radiation oncologist and also collaboratively managed by an otolaryngological and dental team to reduce acute toxicities. After PT, in principle, patients were initially evaluated 4 weeks after treatment completion and subsequently followed up every 2 to 3 months during the first 2 years and at 6-month intervals thereafter. Patients routinely received magnetic resonance imaging or CT scans at each visit. FDG-PET/CT studies were obtained whenever necessary. Acute and late toxicities were assessed with the Common Terminology Criteria for Adverse Events (CTCAE), version 4.0. Acute toxicities were defined as those observed within 90 days of the PT start, whereas late toxicities were those observed after 90 days.

### Statistical Analysis

LC, RC, DMFS, PFS, and OS rates were calculated using the Kaplan-Meier method from the first date of PT. For LC, locally controlled patients were censored at the time of their last follow-up visit or death. RC was defined as the time to first event of lymph node (regional) failure, and patients were censored when they developed local or distant recurrence or died. DMFS was defined as the time to first event among distant recurrences and death. PFS was defined as the time to first event among local, regional, or distant recurrences and death. Living patients without any progression were censored at the time of their last follow-up visit. OS was defined as the time to death from any cause. Living patients were censored at the time of their last follow-up visit. For univariate analyses, log-rank tests were used to compare LC, PFS, and OS among the subgroups listed in Table 3. All statistical tests were 2-tailed/sided, and a *P* value <.05 was considered significant. All statistical analyses were performed with EZR, which is based on R and R-Commander (R Foundation for Statistical Computing, Vienna, Austria) [28].

## Results

### Tumor, Patient, and Treatment Characteristics

**Figure 1.**
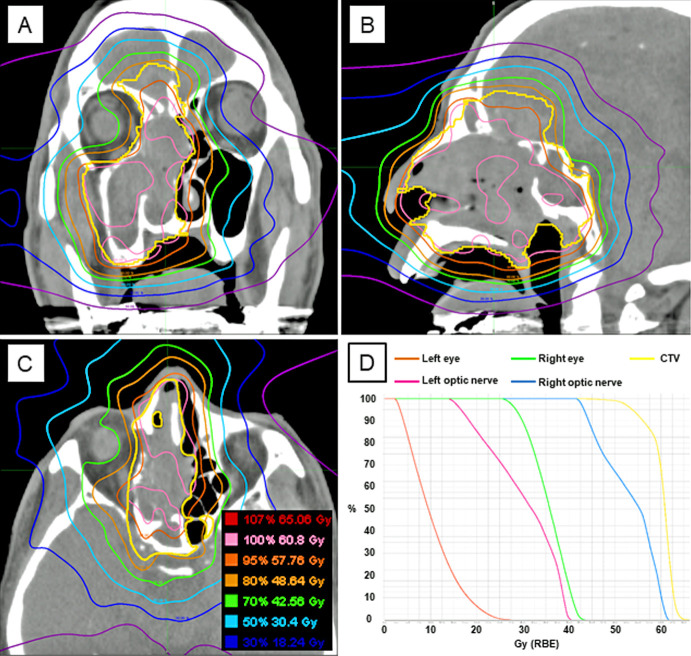
Treatment plan in a representative case of nasal malignant melanoma with intracranial extension (T4bN0M0). Intensity-modulated proton therapy using 3 ports was delivered at 60.8 GyRBE in 16 fractions. The clinical target volume (CTV) was contoured with a yellow line. (A) Coronal image. (B) Sagittal image. (C) Axial image. (D) Dose-volume histograms of the left eye, right eye, left optic nerve, right optic nerve, and CTV.

A total of 62 eligible patients were enrolled in this study. The patient and tumor characteristics are summarized in Table 1, and treatment characteristics are summarized in Table 2. All patients were restaged according to the 8th edition TNM staging system (International Union Against Cancer; UICC, 2017) [29]. We used the classification of malignant melanoma of the upper aerodigestive tract for staging in MM and the classification of nasal cavity and paranasal sinuses for the others. Most patients had T3 or T4 diseases, and orbital and intracranial extension were present in 17 patients (27%) and 16 patients (26%), respectively. Of the 5 patients with a positive lymph node at diagnosis, 3 received PT to the lymph node and primary site simultaneously, and 2 underwent radical neck dissection after PT. Two patients (olfactory neuroblastoma and malignant solitary fibrous tumor [30]) who had no measurable gross tumor received PT with postoperative adjuvant intent. Chemotherapy was administered to 10 patients before and/or after PT, whereas it was not used concurrently with PT. Seven patients, all of whom were MM, adjuvantly received immune checkpoint inhibitors after PT. Regarding PT delivery, IMPT was provided in 42 patients (68%) and SFUD in 20 patients (32%); the median field number was 3 (range, 2-6 beams). The dose distribution of a representative case is shown in Figure 1.

**Table 1.** Patients and tumor characteristics (n = 62).

**Parameter**	**Value**
Age, mean (range), y	70 (23-92)
Sex, n (%)	
Male	41 (66)
Female	21 (34)
Primary site, n (%)	
Nasal cavity or ethmoid sinus	45 (73)
Maxillary sinus	17 (27)
Tumor status, n (%)	
No previous treatment	51 (82)
Postsurgical recurrence	9 (15)
No evidence of gross tumor	2 (3)
Histology, n (%)	
Mucosal melanoma	22 (35)
Squamous cell carcinoma	17 (27)
Adenoid cystic carcinoma	10 (16)
Olfactory neuroblastoma	6 (10)
Carcinosarcoma	2 (3)
Other^a^	5 (8)
T stage, [mucosal melanoma], n (%)	
T1-T2	3 (5)
T3	26 [17], (42)
T4a	12 [3], (19)
T4b	21 [2], (34)
N stage, n (%)	
N0	58 (94)
N1	2 (3)
N2	2 (3)
Orbital extension, n (%)	
Present	17 (27)
Absent	45 (73)
Intracranial extension, n (%)	
Present	16 (26)
Absent	46 (74)

a Includes adenocarcinoma, malignant solitary fibrous tumor, fibrosarcoma, mucoepidermoid carcinoma, and unknown.

**Table 2.** Treatment characteristics.

**Characteristic**	**Value, n (%)**
Proton technique	
SFUD	20 (32)
IMPT	42 (68)
Dose, GyRBE/fraction	
60.8/16	18 (29)
60/15	4 (6)
70.2/26	48 (77)
70/35	1 (2)
65/26	1 (2)
Chemotherapy	
Pre-PT	5 (8)
Post-PT	1 (2)
Pre-PT and post-PT	4 (6)
None	52 (84)
Immune checkpoint inhibitor	7 (32)^a^

a Denominator is the number of mucosal melanoma patients (n = 22).

**Abbreviations:** SFUD, single-field uniform dose; IMPT, intensity-modulated proton therapy; PT, proton therapy.

**Table 3.** Results of log-rank tests for prognostic factors.

**Factor**	**Patient number**	**2-year LC, %**	***P***	**2-year PFS, %**	***P***	**2-year OS, %**	***P***
Age, y			.17		.24		.35
<70	30	91		61		75	
≥70	32	93		39		78	
Sex			.58		.89		.66
Male	41	91		52		72	
Female	21	95		49		83	
Primary site			.71		.82		.53
Nasal cavity or ethmoid sinus	45	95		52		81	
Maxillary sinus	17	83		46		67	
Histology			.64		.76		.021
Mucosal melanoma	22	95		47		81	
Squamous cell carcinoma	17	92		55		67	
Adenoid cystic carcinoma	10	90		46		100	
Olfactory neuroblastoma	6	100		67		100	
Other^a^	7	67		38		29	
Mucosal melanoma vs others			.49		.34		.65
T category			.85		.28		.32
T1-T3	29	96		63		83	
T4	33	88		41		71	
Proton technique			.27		.43		.46
SFUD	20	95		44		74	
IMPT	42	91		57		78	
Chemotherapy^b^			1.0		.17		.74
Yes	9	88		67		78	
No	31	92		48		73	
Immune checkpoint inhibitor^c^			.26		.69		.10
Yes	7	100		71		100	
No	15	93		36		75	

a Includes carcinosarcoma (n = 2), adenocarcinoma (n = 1), malignant solitary fibrous tumor (n = 1), fibrosarcoma (n = 1), mucoepidermoid carcinoma (n = 1), and unknown (n = 1).

b Patients with tumors other than mucosal melanoma were evaluated.

c Patients with mucosal melanoma were evaluated.

**Abbreviations:** LC, local control; PFS, progression-free survival; OS, overall survival; SFUD, single-field uniform dose; IMPT, intensity-modulated proton therapy.

### Locoregional Control and Survival

**Figure 2.**
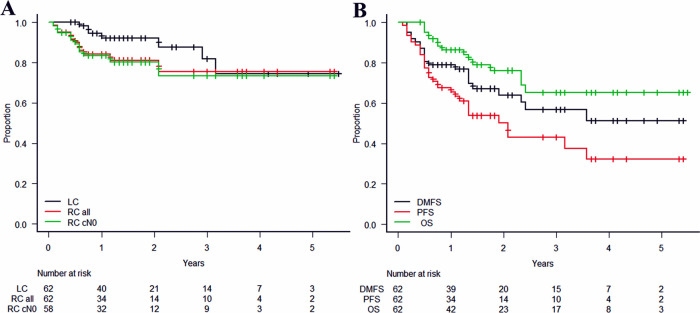
Kaplan-Meier curves: (A) local control (LC) and regional control (RC) for all patients and RC for cN0 patients; (B) distant metastasis-free survival (DMFS), progression-free survival (PFS), and overall survival (OS).

The median follow-up time was 17 months (range, 6-66 months) for all patients and 21.5 months (range, 6-66 months) for survivors. Seven patients were lost to follow-up. LC, RC, DMFS, PFS, and OS curves are shown in Figure 2. The 2-year rates of LC and RC for all patients and RC for cN0 patients were 92% (95% confidence interval [CI], 80%-97%), 81% (95% CI, 67%-90%), and 80% (95% CI, 66%-89%), respectively. The 2-year rates of DMFS, PFS, and OS were 64% (95% CI, 49%-76%), 50% (95% CI, 35%-64%), and 76% (95% CI, 61%-86%), respectively. A total of 15 deaths were observed; 11 were due to distant metastasis, 1 to local recurrence, and 3 to other causes. The initial recurrence patterns in 28 patients were local recurrence in 6 patients, regional lymph node metastasis in 8 patients, distant metastasis in 7 patients, meningeal dissemination in 2 patients, and simultaneous metastases to regional lymph nodes and other sites in 5 patients. Table 3 shows the results of the univariate analysis of prognostic factors for LC, PFS, and OS. We found histology to be significantly associated with OS; patients with adenoid cystic carcinoma and those with olfactory neuroblastoma had a 2-year OS of 100%. However, there were no other notable factors, and multivariate analysis was not performed.

### Acute Toxicity and Late Toxicities

Treatment-related acute and late toxicity profiles are listed in Table 4. Neither acute grade ≥4 toxicities nor late grade 5 toxicities were observed. All patients completed the planned course of PT without interruptions due to toxicities. Acute toxicities consisted mainly of dermatitis and mucositis. Grade 3 acute toxicities included mucositis in 4 patients (6%) and dermatitis in 1 patient (2%). Regarding late toxicities, a total of 16 grade ≥3 toxicities were observed in 12 patients (19%). Two patients experienced grade 3 sinus disorder, and both were treated with outpatient surgical removal of nasal adhesion. One patient experienced grade 3 watering eye and underwent nasolacrimal duct surgery. One patient who experienced grade 3 soft-tissue infection was hospitalized. The profiles of ocular and visual late toxicities are detailed in Table 5. In principle, patients with tumors involving or near the optic nerve were informed of and gave consent to visual impairments after PT, giving priority to coverage of the target rather than sparing the optic nerve. Nine patients experienced visual impairment; the most common cause was cataract. One patient developed unilateral blindness 34 months after PT because of grade 4 optic neuropathy.

**Table 4.** Acute and late toxicities.

**Toxicity**	**Grade 2, n (%)**	**Grade 3, n (%)**	**Grade 4, n (%)**
Acute			
Dermatitis	16 (26)	1 (2)	0
Mucositis	23 (37)	4 (6)	0
Eye pain	2 (3)	0	0
Otitis media	2 (3)	0	0
Otitis externa	2 (3)	0	0
Late			
Visual impairment	4 (6)^a^	10 (16)^b^	1 (2)^b^
Watering eye	1 (2)	1 (2)	0
Dermatitis	2 (3)	0	0
Sinus disorder	31 (50)	2 (3)	0
Epistaxis	2 (3)	0	0
Soft-tissue infection	1 (2)	1 (2)	0
Osteonecrosis	2 (3)	0	0
Otitis media	6 (10)	1 (2)	0
Brain injury	0	0	0
Trismus	0	0	0

a Grade 2 toxicities consisted of dry eye (n = 1), blurred eye (n = 1), cataract (n = 1), and retinopathy (n = 1).

b Grade ≥3 toxicities are detailed in Table 5.

**Table 5.** Profile of grade ≥3 ocular and visual late toxicities.

**Patient**	**Age/ sex**	**Primary site**	**Histology**	**T stage**	**Orbital extension**	**Dose, GyRBE/Fr**	**Cataract**	**Onset, mo**	**Other toxicity**	**Onset, mo**
1	73/M	NC (B)	SCC	T4b	Yes	70.2/26	No		Optic nerve disorder (R) (Gr 4)^a^	34
2	28/M	NC (R)	ON	T4b	No	70/35	No		Retinopathy (R)	10
3	90/F	NC (L)	MM	T4a	No	60.8/16	No		Retinal vascular disorder (L)	13
4	44/F	NC (L)	ON	T3	No	70.2/26	Yes (L)	33	Retinal vascular disorder (L)	33
5	55/M	MS (R)	SCC	T4a	Yes	70.2/26	Yes (R)	29	Optic nerve disorder (R)	36
6	60/M	NC (R)	CS	T4a	No	70.2/26	Yes (R)	33		
7	78/F	MS (L)	ACC	T4b	No	70.2/26	Yes (L)	38		
8	37/M	MS (L)	SCC	T3	No	70.2/26	Yes (L)	35		
9	79/F	NC (L)	MM	T3	No	60/15	Yes (B)	19		

a Grade 4 toxicity was only observed in patient 1 of optic nerve disorder; all other toxicities were grade 3.

**Abbreviations:** Fr, fraction; M, male; NC, nasal cavity; B, bilateral; SCC, squamous cell carcinoma; R, right; ON, olfactory neuroblastoma; F, female; L, left; MM, mucosal melanoma; MS, maxillary sinus; CS, carcinosarcoma; ACC, adenoid cystic carcinoma.

## Discussion

This study investigated the clinical outcomes of SSPT for sinonasal malignant tumors. The population of the present study consisted of heterogeneous histologic subtypes. However, the treatment strategies were relatively uniform; the majority of the patients received SSPT of 70.2 GyRBE in 26 fractions (60 GyRBE in 15 fractions or 60.8 GyRBE in 16 fractions for MM) in a definitive setting without systemic therapy. As a result, for the primary endpoint, we obtained a 2-year LC of 92% for all patients analyzed. Regional lymph node metastasis was the most common initial failure pattern. In addition, severe toxicities other than the expected ocular- and visual-related toxicities were limited.

Previous reviews summarized the superiority of PT for sinonasal tumors over conventional photon therapy [14, 15]. Neurologic toxicity after PT should be concerned, but the clinical benefits of PT might outweigh those of photon therapy in almost every respect except for the current cost disparity. In comparing PT delivery systems, a dosimetric comparison study indicated that PBS provided better dose concentration to the target than PSPT while sparing the organ at risk (especially the skin and optic pathway) [17]. To the best of our knowledge, only 1 recent study has compared them in a clinical setting and reported better LC after PBS [20]. (Note: the aforementioned 2 studies [17, 20] used the term “IMPT” to refer to plans with PBS. To simplify, we reworded their “IMPT” to PBS here and regarded it as almost the same as SSPT.) Although reports about SSPT for sinonasal tumors are extremely rare so far, there are some published reports about PSPT that can serve as a historical control [31–33]. The 2-year LC and OS rates from these reports ranged from 35% to 86% and 47% to 68%, respectively. Our results of 2-year LC and OS (92% and 76%, respectively) seemed to be comparatively favorable. However, care is needed when comparing these data because their analyzed population characteristics and treatment strategies ranged quite widely and differed from ours. In particular, evaluation of the contribution to OS in studies that include heterogeneous histologic subtypes may be difficult. Indeed, our results showed that the PFS and OS rates varied greatly among histologic subtypes (Table 3).

We observed low rates of severe toxicities in both acute and late phases. Possible explanations for this include not only the use of SSPT but also the additional device, called PSAS, used in our institution. The PSAS consists of an energy absorber and collimator, which creates sharp fall-off of the lateral penumbras. This device could help to improve the dose distribution, especially in shallow regions. We previously reported the dosimetric advantages and positive effects on toxicities in patients with head and neck tumors [26, 27]. In addition, intervention by an otolaryngological and dental team from the beginning through to post-PT may have contributed to reducing toxicities. Of those with ocular and visual toxicities, 9 patients (15%) experienced grade ≥3; 4 of them had only cataract. As radiation-induced toxicity can severely degrade patients' quality of life, we should attempt to minimize any unnecessary dose to the visual organs. In some cases, however, high doses were intentionally delivered to these organs to prioritize target coverage under adequate informed consent. It is important in clinical practice to collect evidence and give patients accurate information about these toxicities, including incidence, time of onset, and predictive factors. The time to onset of radiation-induced optic neuropathy was reported to range from 5 to 58 months after PT, and associations with diabetes mellitus, age, maximum dose, and hypertension were suggested [34, 35]. We recognized that our data in Table 5 were not sufficient enough, but we hope it will be of some help to future clinical practice of SSPT.

This study had several limitations. First, this was a retrospective study with a relatively small and heterogeneous population, so the inherent bias is considerable. A smaller set of histologic subtypes would allow a more accurate evaluation of efficacy. However, we included any types of tumor because we intended to evaluate the safety among more patients. Further larger-scale studies should be conducted for each histologic subtype. Another limitation was the short follow-up duration; a median follow-up of 17 months is not sufficient to evaluate both efficacy and toxicity.

We are convinced that SSPT is a leading treatment modality for sinonasal tumors. Nevertheless, it should be noted that SSPT, especially IMPT, remains challenging because of various uncertainties related to physical and biological issues [36]. In addition, the optimal combination with other treatment modalities, including surgery, chemotherapy, and immunotherapy, remains an important issue for discussion. SSPT can be theoretically advantageous not only in the definitive setting but also in postoperative salvage and (neo)adjuvant settings. Also, the low incidence of toxicity should permit concurrent use of systemic therapy. Moreover, synergistic effects with systemic therapy may produce positive outcomes [37, 38]. While the results of the current study came from a population consisting mainly of the definitive setting without systemic therapy, further investigations will hopefully reveal the contributions of SSPT in other situations.

## Conclusion

We demonstrated promising results of SSPT for sinonasal malignant tumors with respect to both efficacy and safety; the 2-year LC rate of 92% irrespective of tumor histology was especially remarkable. SSPT can theoretically result in fewer toxicities than other RT modalities, and additional efforts could lead to further improvements. However, several unanswered questions remain regarding SSPT itself. More evidence from further studies is required to establish its status as a standard treatment for sinonasal malignant tumors.
